# Multiplicative Error Analysis: A Scale-Invariant View of TDDFT Two-Photon Absorption Benchmarks

**DOI:** 10.1021/acs.jctc.6c01476

**Published:** 2026-10-18

**Authors:** Dávid Mester, Mihály Kállay

**Affiliations:** † Department of Physical Chemistry and Materials Science, Faculty of Chemical Technology and Biotechnology, Budapest University of Technology and Economics, Műegyetem rkp. 3., Budapest H-1111, Hungary; ‡ HUN-REN−BME Quantum Chemistry Research Group, Műegyetem rkp. 3., Budapest H-1111, Hungary; § MTA−BME Lendület Quantum Chemistry Research Group, Műegyetem rkp. 3., Budapest H-1111, Hungary

## Abstract

Two-photon absorption (2PA) strengths span several orders of magnitude, yet time-dependent density functional theory (TDDFT) benchmark studies have commonly relied on raw-scale mean absolute errors and linear regression statistics. Such statistics can be dominated by the most intense transitions and do not distinguish systematic multiplicative bias, typical fold error, dynamic range reproduction, and rank preservation. Here, published TDDFT 2PA benchmarks based on coupled-cluster references are reanalyzed by using a scale-invariant, multiplicative framework. For the most widely used benchmark set [*J. Chem.* *Theory Comput.* 2022, 18, 1046], it is shown that the five strongest transitions, representing only approximately 10% of the data, account for up to 65.5% of the total raw absolute error. Consequently, raw-scale metrics assign disproportionate weight to a small number of intense transitions and may obscure method performance across the remainder of the intensity range. Across several benchmark sets, the analysis further reveals that similar raw-scale errors can arise from qualitatively different error structures. The multiplicative analysis goes beyond previously recognized qualitative trends by resolving them into directly comparable and interpretable components. These results demonstrate that neither individual raw-scale metrics nor their combinations provide a sufficient basis for identifying a reliable or generally recommended functional, and they support a multidimensional, scale-invariant assessment of 2PA benchmarks. The same framework may also be useful for other properties for which multiplicative errors provide a more appropriate description than additive deviations such as oscillator strengths, kinetic and radiative rate constants, and equilibrium constants.

## Graphical abstract

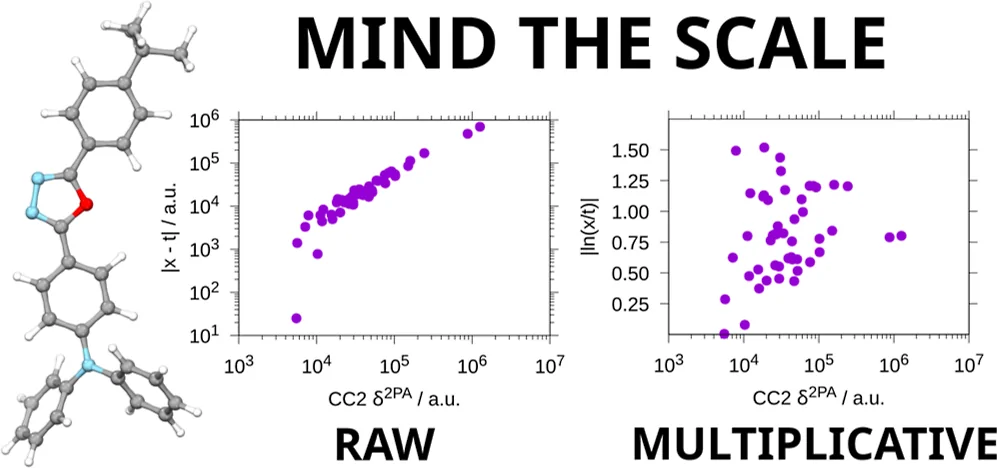

## 1 Introduction

Two-photon absorption (2PA) is a nonlinear optical process in which a molecular system is excited through the simultaneous absorption of two photons. The phenomenon was predicted during the early development of quantum mechanics by Göppert-Mayer^1^ and experimentally demonstrated three decades later by Kaiser and Garrett.^2^ Under typical experimental conditions, the two-photon excitation rate is proportional to the square of the local intensity of light. This nonlinear dependence strongly localizes excitation around the focal volume and suppresses out-of-focus excitation. In biological tissue, near-infrared excitation can additionally reduce scattering and increase penetration depth within suitable optical windows.^3–5^ These properties underpin multiphoton microscopy and bioimaging^3,4^ as well as two-photon polymerization for three-dimensional microfabrication and optical data storage.^6^ Two-photon excitation has also been explored for photodynamic therapy^7^ and in a broad range of nonlinear-optical and photonic materials.^8,9^

The growing range of applications has stimulated the design of chromophores with enhanced and tunable 2PA responses. Early structure–property studies established the importance of extended π conjugation, donor–acceptor substitution, molecular symmetry, and multipolar architectures, and these principles continue to guide the development of two-photon dyes.^8–12^ Nevertheless, the relationship between molecular structure and 2PA intensity is far from straightforward. The electronic transition tensors can be highly sensitive to the excitation character, molecular conformation, environmental effects, and the balance among intermediate-state contributions. Moreover, the spectroscopic 2PA cross section is not a purely electronic quantity: in addition to the electronic transition tensor, it depends on the photon energy, line shape, polarization and orientational averaging, and vibronic effects. Experimentally inferred effective cross sections may also depend on the excitation bandwidth and measurement protocol.^13–17^ Reliable electronic-structure calculations are therefore valuable both for interpreting measurements and for screening new chromophores.

From a theoretical point of view, response theory provides a natural framework for the calculation of multiphoton properties. The 2PA transition strength can be obtained from a residue of the quadratic response function, thereby avoiding an explicit truncation of the sum over intermediate states.^13,18^ Near intermediate-state resonances, damped response theory provides a finite-frequency treatment of otherwise divergent response expressions.^19^ Within wave function theory, coupled-cluster (CC) response methods^20^ offer a systematically improvable hierarchy and have therefore played a central role in establishing reference values. Early formulations provided general expressions for multiphoton transition moments, and applications across the hierarchy demonstrated the importance of electron correlation.^13,21^ Subsequent algorithmic and implementation advances included resolution-of-identity techniques,^22,23^ Cholesky-decomposition-based formulations,^23^ and efficient implementations of higher-level response methods.^24–26^ Together, these developments made 2PA calculations feasible for increasingly large and complex molecular systems and enabled demanding benchmark applications.^27–29^ The main limitation of these approaches is still their steep computational scaling, which restricts the routine use of the most accurate models and makes less expensive methods indispensable for larger molecular systems.

Alternative correlated-response approaches have also been developed. Within the algebraic diagrammatic construction/intermediate-state representation (ADC/ISR) theory, nonlinear response properties can be evaluated using a perturbative hierarchy.^30^ More recent work introduced a general implementation of ADC/ISR response expressions through third order, together with a memory-efficient formulation of the second-order variant, thereby broadening the practical applicability of correlated nonlinear response calculations.^31^

Time-dependent density functional theory (TDDFT) is the most widely used alternative because of its favorable balance between computational cost and applicability. Its linear- and nonlinear-response formulations are well established, and quadratic-response TDDFT has been applied to 2PA for more than two decades.^18,32–36^ The accuracy of the resulting transition strengths, however, depends strongly on the exchange–correlation functional and the character of the excited states. The deficiencies of conventional functionals for charge-transfer excitations^37,38^ are particularly relevant to donor–acceptor chromophores, whereas Rydberg states pose a separate challenge in small-molecule benchmarks and require an appropriate asymptotic potential together with sufficiently diffuse basis sets. Even favorable excitation energies do not guarantee reliable nonlinear transition strengths.^16,24,28,39,40^ Recent 2PA studies have broadened the methodological scope in several distinct directions. These include gauge-invariant current-density meta-generalized gradient approximation (meta-GGA) response theory,^41^ assessments of the Tamm–Dancoff approximation,^42^ reduced-cost TDDFT-based approaches,^43–46^ vibronic and spin–orbit treatments,^17,47^ and Bethe–Salpeter equation calculations.^48,49^ Collectively, these developments improve formal consistency, numerical affordability, or the range of physical effects that can be described while increasing the need for transparent procedures for assessing predictive performance.

Accordingly, 2PA benchmarking has progressed from compact studies of prototypical small molecules and fluorescent-protein chromophores to larger chemically focused series and broad collections spanning diverse molecular sizes and excitation characters.^16,24,27–29,40,42,50–56^ Several benchmark sets have subsequently been revisited with additional functional classes, statistical measures, or response-theory formulations.^41,57,58^ Taken together, these studies show that functional performance depends strongly on the system, the property considered, and even the manner in which the results are evaluated. The increasing size and diversity of the available data sets therefore make the statistical characterization of the errors nearly as important as the choice of the underlying electronic-structure method.

Most 2PA benchmark studies have emphasized raw-scale mean absolute errors (MAEs) and linear-regression slopes, intercepts, or Pearson correlations, whereas a smaller number have additionally reported the mean absolute percentage error (MAPE), direct ratios, relative logarithmic errors, or rank correlations. The former metrics are useful and address different aspects of performance, but their conventional combinations do not provide an unambiguous overall description when the reference values span several orders of magnitude. When absolute residuals increase with the magnitude of the reference value, raw-scale MAEs can become dominated by the most intense transitions, while a high correlation on the original scale primarily reflects a linear association rather than agreement. MAPE is dimensionless but treats over- and underestimations by the same factor asymmetrically. In the broader statistical literature, the properties of percentage errors and the advantages of logarithmic accuracy ratios for positive-valued quantities have been analyzed in detail.^59,60^ Relative logarithmic errors and rank-based measures have already been used in individual 2PA studies,^41,57^ but they have not routinely been combined with measures that separately describe systematic bias, typical and larger multiplicative errors, the reproduction of the overall intensity range, and the preservation of molecular or transition rankings. Moreover, an ordinary correlation coefficient can remain high, even when the predictions are systematically shifted away from the reference values. This motivates the use of agreement measures that assess not only whether the two sets of values vary together but also how closely they follow the ideal relation in which each prediction equals its reference value.^61^

In the present work, we apply a multidimensional, scale-invariant framework for analyzing TDDFT 2PA benchmarks. Four complementary data sets based on CC reference values are reanalyzed using multiplicative bias, fold-error statistics, logarithmic concordance, log–log slopes, and rank correlations. In addition, a bias-dominance index and leave-one-out (LOO) calibration are used to characterize coherent multiplicative shifts. The purpose is not to replace all raw-scale statistics or to prescribe a single functional but to clarify which aspects of predictive performance are measured by the different indicators and to provide a more consistent basis for interpreting results across data sets with different intensity ranges. Although the analysis is demonstrated for 2PA strengths, the same considerations may extend naturally to other properties for which a multiplicative error model is better supported than an additive one.

## 2 Theory

2PA strengths, δ^2PA^, provide a particularly challenging target for benchmarking. In typical benchmark sets, the reference 2PA strengths span several orders of magnitude. This wide dynamic range has an important statistical consequence: numerical errors are often not naturally additive on the original scale. A deviation of, for example, 10 a.u. may be chemically significant for a weak transition but essentially negligible for a very intense one. Similarly, an error of several thousand a.u. may represent only a modest relative deviation for a strong transition but an unacceptable error for a weak transition. Therefore, the absolute deviation on the original scale is not a scale-invariant measure of predictive quality.

This issue is especially relevant when TDDFT results are compared against higher-level CC reference data such as second-order approximate CC singles and doubles (CC2)^62^ or CC singles, doubles, and approximate triples (CC3).^63^ The central question is not merely whether the absolute numerical difference between a calculated 2PA strength and a reference value is small but whether the method predicts the correct order of magnitude, the correct relative trends, and the correct multiplicative scale.

Let *t*_*i*_ > 0 denote the reference 2PA strength for transition *i*, and let *x*_*i*_ > 0 denote the corresponding value predicted by the approximate method. The statistical problem can be made explicit by contrasting additive and multiplicative error models. In an additive model, the calculated value is written as

1 $$x_{i}=t_{i}+e_{i}$$

where the typical size of the raw residual *e*_*i*_ is approximately independent of the magnitude of *t*_*i*_. If this model were appropriate for 2PA strengths, a calculation of similar quality would exhibit residuals of similar absolute size for weak and strong transitions. In contrast, Figure 1 illustrates why this assumption is problematic using a benchmark set comprising 48 dipolar π-conjugated systems, with CAM-B3LYP^64^ results compared against CC2 reference values.^40^

**Figure 1:**
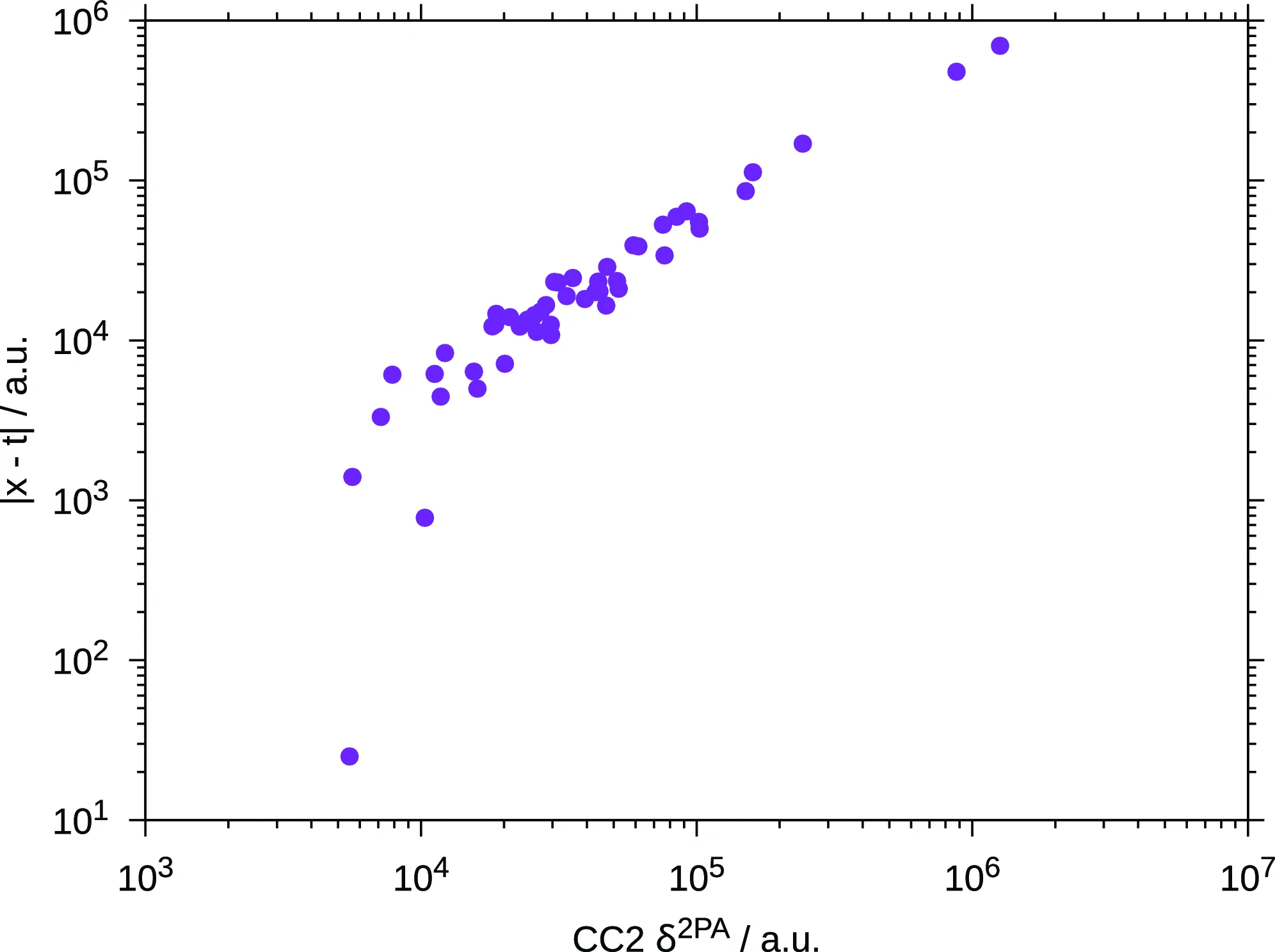
Absolute raw residuals for CAM-B3LYP on the 48-molecule dipolar push–pull chromophore benchmark of ref 40, plotted against the CC2 reference 2PA strengths.

The figure clearly shows that the raw residuals are strongly associated with the reference 2PA strengths: the largest residuals occur predominantly for the largest reference values. This behavior is inconsistent with an additive, roughly homoscedastic error model. In a heterogeneous benchmark set containing both weak and strong transitions, raw-scale error metrics will therefore be dominated by the high-intensity part of the data.

For 2PA strengths, a more natural description is often multiplicative,

$$x_{i}=t_{i}\;\eta_{i}$$ 2

where η_*i*_ is a dimensionless error factor. The raw residual is then *x*_*i*_ – *t*_*i*_ = *t*_*i*_(η_*i*_ – 1), showing that, for a given relative error, the absolute residual is proportional to the magnitude of the reference value. Consequently, even if the relative error remains constant, larger reference values will naturally exhibit larger absolute deviations. This behavior is therefore expected for multiplicative errors and does not necessarily indicate poorer performance for stronger transitions. Accordingly, the logarithmic scale provides a more appropriate basis for error analysis. Under the multiplicative model, $\ln\left(\frac{x_{i}}{t_{i}}\right)=\ln\eta_{i}$ , so the logarithmic residual directly represents the logarithmic fold error, independently of the magnitude of the reference value.

This statistical distinction has direct consequences for conventional benchmark metrics. Under the additive model, a natural summary of the residuals is the MAE,

3 $$MAE=\frac{1}{n}\sum_{i=1}^{n}\left|e_{i}\right|=\frac{1}{n}\sum_{i=1}^{n}\left|x_{i}-t_{i}\right|$$

Although such a quantity is straightforward to interpret on the original scale, it is not scale-invariant when the target variable spans several orders of magnitude and the expected error grows with the magnitude of the reference value. In that case, the MAE is dominated by the largest 2PA strengths in the benchmark set and gives disproportionately less weight to low-intensity transitions. As a result, two methods with very different qualitative behaviors may obtain similar MAE values if their errors are concentrated in different intensity regimes. This does not imply that raw-scale errors are useless: if a specific application focuses on a narrow-intensity range, raw errors can still be informative as supplementary metrics. They should not, however, be the sole basis for comparing methods across a benchmark set spanning orders of magnitude.

A related problem arises for the ordinary Pearson correlation coefficient *r* or for its square *r* ^2^ when it is applied directly to the original 2PA strengths. Throughout this work, raw-scale *r* ^2^ denotes the square of the Pearson correlation coefficient; equivalently, for a single predictor, it is the coefficient of determination from an ordinary least-squares regression of *x*_*i*_ on *t*_*i*_ with a fitted intercept. Raw-scale correlation measures the linear association on a scale where the largest values can have high leverage. Consequently, a method may achieve an apparently favorable raw-scale correlation by reproducing the ordering of the largest 2PA strengths reasonably well while failing systematically for weaker transitions. Conversely, a method that captures the correct multiplicative trends may be penalized if the residuals are not well represented by an additive model on the original scale.

The fundamental quantity for the logarithmic analysis is the logarithmic fold error,

4 $$z_{i}=\ln\left(\frac{x_{i}}{t_{i}}\right)=\ln\left(\frac{x_{i}}{c}\right)-\ln\left(\frac{t_{i}}{c}\right)$$

where *c* is any positive scaling constant with the same physical units as the reported 2PA strengths, for example, *c* = 1 a.u. of 2PA strength. This form makes explicit that only dimensionless ratios are used inside the logarithms; the value of *z*_*i*_ is therefore independent of the particular choice of *c*. The logarithmic fold error measures the error as a multiplicative factor rather than as an additive difference. If *z*_*i*_ = 0, the prediction is exact. If *z*_*i*_ = ln 2, the method overestimates the reference value by a factor of 2. If *z*_*i*_ = – ln 2, the method underestimates the reference value by a factor of 2. Thus, positive and negative errors of equal magnitude correspond to reciprocal over- and underestimation factors, which is the desired symmetry for multiplicative errors. The multiplicative description is appropriate when the target quantity is strictly positive and relative errors are the relevant measure of accuracy. It is not defined for zero or sign-changing quantities and becomes increasingly sensitive for very small values, such as effectively forbidden transitions. When a fixed absolute deviation is the scientifically relevant tolerance, raw-scale error measures remain the more natural choice. Figure 2 shows the same CAM-B3LYP data after transforming the residuals to the logarithmic scale.

**Figure 2:**
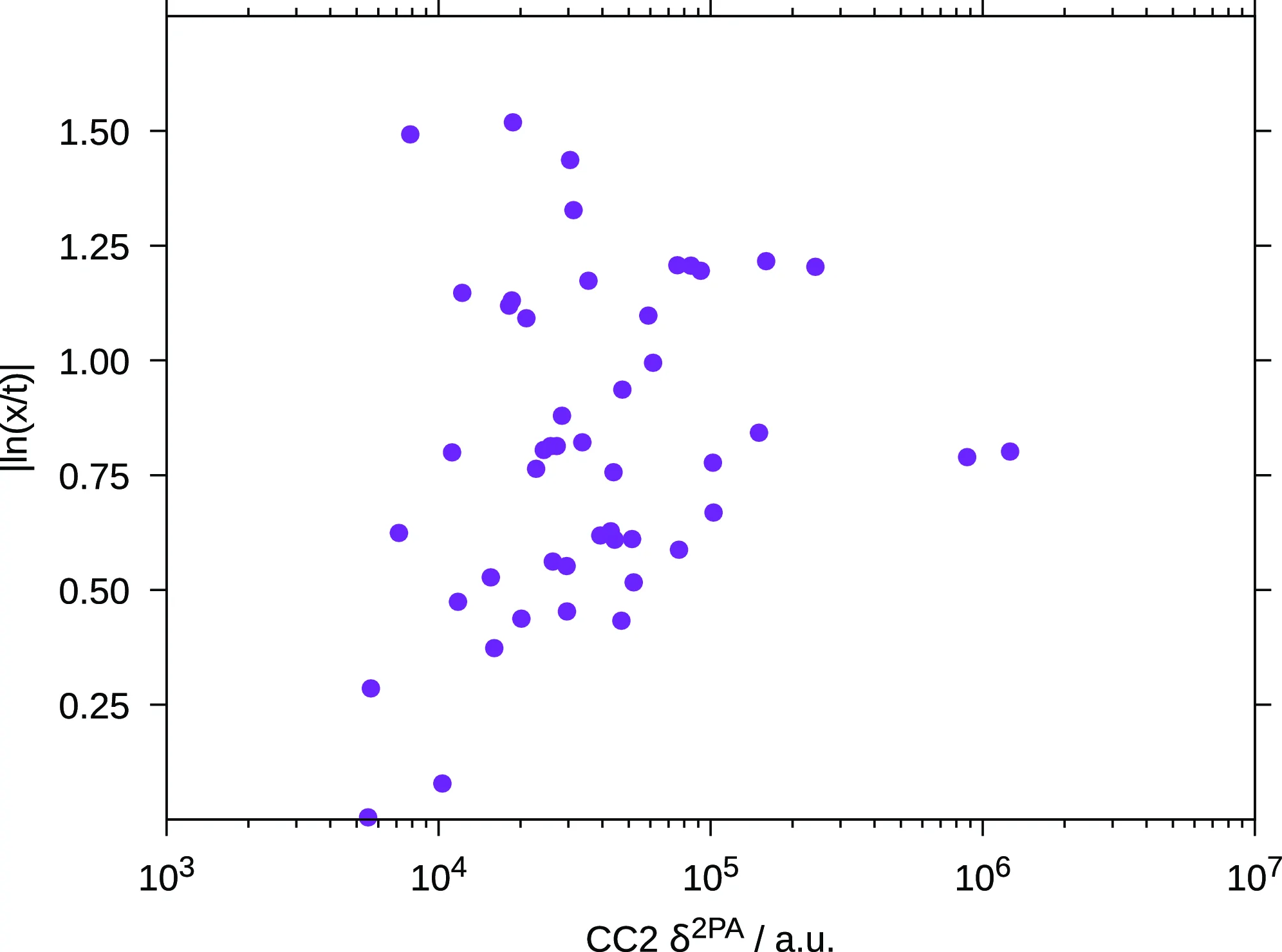
Absolute logarithmic residuals for CAM-B3LYP on the 48-molecule dipolar push–pull chromophore benchmark of ref 40, plotted against the CC2 reference 2PA strengths.

Although the logarithmic transformation does not completely eliminate heteroscedasticity, it strongly reduces the dominant scale dependence observed for the raw residuals and makes the errors more naturally comparable across weak and strong transitions; the proposed metrics do not require a homoscedastic log-error model. For the illustrative CAM-B3LYP data on this benchmark, the Spearman correlation between the reference strength and residual magnitude decreases from 0.961 for |*x*_*i*_ – *t*_*i*_| to 0.268 for | ln­(*x*_*i*_/*t*_*i*_)|, providing a quantitative measure of this reduction. Because residual magnitudes do not retain the direction of the error, the corresponding signed log residuals are shown in Figure S1 of the Supporting Information. They are predominantly negative, consistent with the systematic underestimation by CAM-B3LYP, but do not exhibit the pronounced scale dependence seen for the raw residuals.

Having established *z*_*i*_ as the basic residual on the logarithmic scale, one can now define summary metrics that describe complementary aspects of method performance. First, the signed average logarithmic error can be transformed back to the multiplicative scale to obtain the geometric mean bias,^59^

5 $$B=\exp\left[\frac{1}{n}\sum_{i=1}^{n}z_{i}\right]=\exp\left[\frac{1}{n}\sum_{i=1}^{n}\ln\left(\frac{x_{i}}{t_{i}}\right)\right]$$

The quantity *B* is the geometric mean of the ratios *x*_*i*_/*t*_*i*_. It measures the systematic multiplicative bias of the method. A value of *B* = 1 indicates no geometric mean multiplicative bias. Values larger than one indicate systematic overestimation, while values smaller than one indicate a systematic underestimation. For example, *B* = 2 means that, on the geometric mean, the method predicts twice the reference 2PA strengths, whereas *B* = 0.5 means that its predictions are half of the reference values, corresponding to underestimation by a factor of 2.

To quantify the typical unsigned multiplicative error, the geometric mean fold-error factor^59^ is defined as

6 $$F=\exp\left[\frac{1}{n}\sum_{i=1}^{n}\left|z_{i}\right|\right]=\exp\left[\frac{1}{n}\sum_{i=1}^{n}\left|\ln\left(\frac{x_{i}}{t_{i}}\right)\right|\right]$$

This metric is always greater than or equal to one. This can be interpreted as the typical multiplicative error factor of the method. For instance, *F* = 1.5 means that the predicted 2PA strengths differ from the reference values by a typical factor of 1.5, irrespective of whether the individual deviations are over- or underestimations. Unlike the MAE, this metric weighs relative deviations equally across the entire intensity range. An error by a factor of 2 contributes the same amount whether the reference value is small or large.

A closely related dimensionless quantity is the mean absolute percentage error,

7 $$MAPE=\frac{100}{n}\sum_{i=1}^{n}\left|\frac{x_{i}-t_{i}}{t_{i}}\right|=\frac{100}{n}\sum_{i=1}^{n}\left|\exp\left(z_{i}\right)-1\right|$$

Although MAPE is also scale-independent, it is not symmetric for over- and underestimations by the same factor. For example, a factor-of-two overestimation gives |*x*_*i*_/*t*_*i*_ – 1| = 1, whereas a factor-of-two underestimation gives |*x*_*i*_/*t*_*i*_ – 1| = 0.5. In contrast, the corresponding logarithmic residuals are + ln 2 and – ln 2, which have the same absolute magnitude. Thus, log-fold errors treat over- and underestimations by the same factor equivalently, making them more natural for 2PA strengths spanning several orders of magnitude.

In addition to *F*, it is useful to report a high-percentile fold-error factor. For a quantile level *p*, this can be defined as

8 $$F_{p}=\exp\left[Q_{p}\left(|\ln\left(\frac{x_{i}}{t_{i}}\right)|\right)\right]$$

where *Q*_*p*_ denotes the *p*-quantile of the distribution of absolute log fold errors. Throughout this work, *Q*_*p*_ is evaluated using Hyndman and Fan’s Definition 7, corresponding to linear interpolation between adjacent order statistics.^65^ This metric answers a practically important question: for approximately 100*p* % of the benchmark cases, by, at most, what multiplicative factor does the method deviate from the reference? For example, *F*_0.9_ = 3 means that approximately 90% of the predictions differ from their corresponding reference values by no more than a factor of 3. While *F* describes the typical performance, *F*_*p*_ provides a descriptive upper-tail summary for the best 100*p* % of the benchmark cases. Because it is a percentile-based statistic, *F*_*p*_ does not strongly penalize the most extreme outliers in the worst 100­(1 – *p*)% of cases. This is often desirable when the aim is to characterize robust performance rather than worst-case behavior, but it also means that *F*_*p*_ should be complemented by explicit inspection of large outliers. In general, we use *p* = 0.9 for the high-percentile error summary. For a few benchmark sets with a small number of transitions, *p* = 0.8 is applied. The percentile used is specified in each table.

Two additional diagnostics are useful for interpreting strongly asymmetric multiplicative errors. First, the relationship between *B* and *F* can be used to quantify the extent to which the error is dominated by a systematic multiplicative shift. Since |⟨*z*⟩| ≤ ⟨|*z*|⟩, the bias-dominance index is defined as $S=\frac{\left|\ln B\right|}{\ln F}$ and satisfies 0 ≤ *S* ≤ 1 for *F* > 1. Values close to unity indicate that the logarithmic errors are predominantly one-sided, whereas values close to zero indicate that positive and negative errors largely cancel in the signed logarithmic mean. For a perfect method with *F* = 1, the index is not defined because bias dominance is not meaningful. As a complementary descriptive quantity, we also report the fraction of underestimated cases, defined as the proportion of observations for which *x*_*i*_ < *t*_*i*_.

A strongly bias-dominated error distribution may be suitable for a simple multiplicative calibration. To assess this possibility without using the observation being predicted, we adapt the LOO cross-validation principle^66^ to multiplicative bias correction. For each omitted transition *i*, the geometric mean bias is estimated from the remaining *n* – 1 observations,

9 $$B_{\left(-i\right)}=\exp\left[\frac{1}{n-1}\sum_{j\neq i}\ln\left(\frac{x_{j}}{t_{j}}\right)\right]$$

and the held-out prediction is corrected according to *x*_*i*_^cal^ = *x*_*i*_/*B*_(‑*i*)_. The LOO-calibrated fold-error factor is then calculated exclusively from the assembled held-out predictions,

10 $$F_{LOO}=\exp\left[\frac{1}{n}\sum_{i=1}^{n}\left|\ln\left(\frac{x_{i}^{cal}}{t_{i}}\right)\right|\right]$$

A substantial reduction from *F* to *F*_LOO_ indicates that the errors within the benchmark contain a coherent multiplicative offset that is reduced by bias correction. Because the calibration factor is re-estimated for each omitted observation, the assembled LOO predictions are not obtained through a single uniform rescaling of the original predictions. Consequently, some relationships among the predictions may change, whereas a single multiplicative factor estimated from an independent calibration set would leave them unchanged. Accordingly, *F*_LOO_ is used here as an internal residual-structure diagnostic and is not interpreted as evidence that a calibration factor transfers to chemically distinct systems. Owing to their greater sensitivity to sample size and their role as secondary diagnostics, the bias-dominance index *S* and the LOO-calibrated fold-error factor are discussed only for the 48-system push–pull chromophore benchmark,^40^ which provides the widest functional coverage among the selected data sets.

In addition to fold-error statistics, overall agreement on the logarithmic scale can be assessed using Lin’s concordance correlation coefficient.^61^ With *u*_*i*_ = ln­(*x*_*i*_/*c*) and *v*_*i*_ = ln­(*t*_*i*_/*c*), Lin’s coefficient is

11 $$\rho_{c}=\frac{2s_{uv}}{s_{u}^{2}+s_{v}^{2}+{\left(\mathrm{u̅}-\mathrm{v̅}\right)}^{2}}$$

Here, *u̅* and *v̅* are the sample means, while *s*_*u*_^2^, *s*_*v*_^2^, and *s*_*uv*_ are evaluated as sample moments using the denominator *n*, consistently in all terms of ρ_*c*_. Because both *u*_*i*_ and *v*_*i*_ shift by the same constant when *c* is changed, ρ_*c*_ is independent of the arbitrary choice of *c*. Unlike an ordinary correlation coefficient, Lin’s concordance correlation coefficient also penalizes systematic deviations from the identity line. It therefore provides a compact measure of multiplicative agreement between calculated and reference 2PA strengths on the logarithmic scale.

A complementary view of numerical agreement is obtained by fitting a log–log calibration line to the same dimensionless logarithms,

12 $${û}_{i}=a_{\log}+b_{\log}v_{i}$$

where ${û}_{i}$ denotes the fitted value of *u*_*i*_, and *a*_log_ and *b*_log_ are obtained by ordinary least-squares regression of *u*_*i*_ on *v*_*i*_. For an ideal method, one would obtain

13 $$a_{\log}=0,\;b_{\log}=1$$

and the fitted calibration line would coincide with the identity line on the logarithmic scale. For a fixed choice of *c*, the intercept *a*_log_ measures a systematic vertical offset of the calibration line, while the slope *b*_log_ measures how well the method reproduces the dynamic range of the reference data. The intercept should not be compared across analyses that use different units unless *b*_log_ = 1, because changing *c* changes *a*_log_ when the slope differs from unity. If *b*_log_ < 1, the method compresses the range of 2PA strengths; if *b*_log_ > 1, the method exaggerates the dynamic range. After the average offset has been accounted for, compression usually means that weak transitions are predicted too high relative to strong transitions, whereas exaggeration means the opposite. Thus, the log–log slope provides information that is not captured by a single average error metric. It should be interpreted as a calibration diagnostic rather than as a symmetric measure of agreement, because ordinary least-squares regression treats the reference values as the predictor and the calculated values as the response. The resulting *b*_log_ is therefore conditional on the chosen reference coordinate; uncertainty in the reference values may also affect the fitted slope and should not be attributed solely to the approximate method.

Another relevant aspect of performance is whether the method preserves the ordering of the 2PA strengths. This is particularly important when the goal is to screen chromophores, identify strong two-photon absorbers, or compare relative intensities within a series. For this purpose, a rank-based correlation coefficient is more appropriate than a raw-scale Pearson correlation. We therefore consider

14 $$\rho_{rank}=corr\left(rank\left(x_{i}\right),rank\left(t_{i}\right)\right)$$

When two or more observations have identical values, each is assigned the average of the ranks that they would otherwise occupy. The correlation of these ranks corresponds to the Spearman rank correlation coefficient.^67^ A value close to one indicates that the method correctly ranks the transitions or molecules according to their 2PA strength, even if there is a systematic multiplicative bias. A low rank correlation indicates that the method fails to reproduce the relative ordering of the benchmark data, which is a serious limitation for screening applications.

However, rank preservation and absolute accuracy are different properties. A method can have an excellent rank correlation and still be quantitatively poor if all values are systematically too large or too small, or if the dynamic range is compressed or exaggerated. Conversely, a method may have a favorable average absolute error on a particular benchmark set while failing to provide a reliable ranking across molecules or transitions. This distinction is important when identifying a “best” density functional. Benchmark studies that rank methods only by MAE, raw-scale *r* ^2^, or a similarly limited single statistic may confound several different aspects of performance: systematic bias, typical fold error, dynamic-range reproduction, rank preservation, and outlier robustness. A meaningful benchmark comparison should therefore report multiple, complementary metrics rather than reduce the assessment to a single raw-scale score.

In summary, the role of the main metrics used in the present analysis is presented in Table 1.

**Table 1:** Definitions and Practical Interpretation of the Benchmark Metrics Used in the Logarithmic-Scale Analysis

metric	name	practical question answered
*B*	Geometric mean bias	By what factor are the predictions systematically shifted relative to the references?
*F*	Geometric mean fold-error factor	By what typical multiplicative factor do the predictions differ from the references?
*F*_*p*_	High-percentile fold-error factor	For 100p % of cases, by, at most, what factor does the method deviate from the reference?
*S*	Bias-dominance index	Is the multiplicative error dominated by a coherent one-sided bias or by cancellation?
*F*_LOO_	LOO-calibrated fold-error factor	How much multiplicative error remains after cross-validated bias correction?
ρ_*c*_	Lin’s concordance correlation coefficient	How close are the predictions to the references on the log scale, including bias and scale errors?
*b*_log_	Log–log calibration slope	Does the method reproduce the dynamic range, or does it compress or exaggerate it?
ρ_rank_	Spearman rank correlation	Does the method preserve the ordering of the transitions?

The direct measures of numerical predictive accuracy in this framework are *F* and *F*_p_, because they quantify unsigned deviations from the reference values on the multiplicative scale. The index *S* is instead a diagnostic of error structure, while *F*_LOO_ measures the extent to which a systematic multiplicative offset can be removed by cross-validated calibration. The metric most directly associated with dynamic-range reproduction is *b*_log_, since it measures whether the dynamic range of the reference data is reproduced, compressed, or exaggerated. In contrast, ρ_rank_ measures only the preservation of ordering. Lin’s concordance coefficient ρ_*c*_ should therefore be viewed as an overall log-scale agreement metric rather than as a pure trend metric, because it penalizes both lack of association and systematic departures from the identity line. The Python script developed to calculate the statistical metrics is archived in the Zenodo repository associated with this work.^68^

This scale-invariant interpretation can facilitate comparisons among different benchmark sets. Raw-scale quantities such as MAE depend strongly on the numerical range and composition of the data set; a set containing several very intense transitions will naturally produce much larger absolute errors than a set dominated by weak transitions, even if the multiplicative accuracy of the method is similar. In contrast, all metrics summarized in Table 1 are dimensionless and therefore facilitate comparisons across benchmark sets with different intensity ranges, although their values may still depend on several factors, including the chemical composition and size of the benchmark set, the reference method, the basis set, etc.

## 3 Benchmark Sets

Over the past two decades, 2PA benchmarking has developed into a rich and increasingly diverse field. Early studies focused on a few prototypical small molecules^24^ and fluorescent-protein chromophores,^16,50,52^ whereas subsequent work introduced chemically coherent benchmark series comprising organoboron chelates,^51^ push–pull chromophores,^40^ coumarin dyes,^54^ dipyrromethene (DIPY) derivatives,^56^ modified nucleobases,^42^ fluorescent dyes,^55^ rhodopsin chromophore models,^27,53^ and retinal models.^29^ The largest available collections contain dozens of molecules or transitions and cover broad ranges of molecular size, excitation character, chemical environment, and 2PA intensity.^28,40^ Several benchmark sets have also been revisited using additional functional classes, statistical measures, and response-theory formulations, further increasing their value for methodological assessment.^41,57,58^

The aim of the present work is not to provide an exhaustive reanalysis of every published benchmark but to examine the consequences of raw-scale and multiplicative error measures for a small number of selected benchmark data sets. We therefore focus on four complementary sets. These were selected because they contain comparatively large numbers of transitions, provide a particularly well-defined test within a chemically coherent molecular family, or conversely span a broad range of molecular and excitation types. As will be shown below, the scale-invariant analysis largely preserves the main qualitative conclusions reached in the original studies. Its principal advantage is therefore not to overturn the existing literature but to place those conclusions on a more transparent and quantitatively comparable basis while also enabling additional conclusions that cannot be obtained from raw-scale analyses alone.

Only benchmark studies based on CC reference data are included, thereby avoiding the heterogeneous experimental conditions and line-shape assumptions associated with measured cross sections. These reference data are not assumed to be exact or of uniform quality: in particular, CC2 and CC3 should not be regarded as equally accurate, and differences between benchmark sets may partly reflect the level and basis set of the underlying reference calculations. Small numerical differences between functionals should therefore not be overinterpreted. The analysis is carried out for microscopic 2PA strengths, δ^2PA^, because these are the response quantities directly delivered by electronic-structure calculations. Cross sections, σ^2PA^, are closer to experiment but also contain excitation-energy and line-shape factors. The present work is intended primarily as a statistical reanalysis rather than as a source of new benchmark data. Accordingly, the quantities analyzed here were extracted from published data. The only exception is the small-molecule benchmark,^28^ for which new gauge-consistent TDDFT strengths were calculated in the length gauge to replace the previously compared velocity-gauge values. The four selected benchmark sets and the related studies are briefly summarized below.

Chołuj and co-workers^40^ introduced a benchmark set of 48 push–pull π-conjugated molecules featuring low-lying intramolecular charge-transfer transitions in donor–acceptor systems. Using CC2 as the reference, they assessed BLYP,^69,70^ PBE,^71^ B3LYP,^72^ PBE0,^73^ CAM-B3LYP, LC-BLYP,^74^ and tuned LC-BLYP.^75^ For the complete benchmark set, the original statistical analysis was limited to linear-regression quantities, namely, slopes, intercepts, and raw *r* ^2^ values. Because the reference values span several orders of magnitude, this benchmark provides one of the clearest motivations for the logarithmic error analysis adopted in the present work.

Grotjahn and Furche^41^ subsequently examined a complementary formal aspect of the same benchmark, namely, gauge invariance in meta-GGA response calculations. In conventional meta-GGA response theory, the dependence on the kinetic-energy density introduces a formal gauge-origin problem, whereas the corresponding current-density meta-GGA formulation restores gauge invariance. Throughout this work, gauge-invariant current-density meta-GGA variants are denoted by the prefix “*c*” (e.g., cM06-2X). The performance of the methods was assessed mainly through relative logarithmic errors, which provide a scale-independent description.

The same benchmark was later used by Ahmadzadeh and co-workers^57^ to assess SCAN-type,^76,77^ Minnesota,^78,79^ MPWB1K,^80^ BB1K,^81^ and related meta-GGA functionals.^82–84^ Their analysis considered raw-scale linear correlations, Spearman rank correlations, and relative-error statistics. In a subsequent comment, Grotjahn and Furche^58^ pointed out that these meta-GGA calculations employed the conventional gauge-variant response formulation and showed that its numerical effect, although small on average, can be appreciable for individual transitions. Although highly favorable raw-scale correlations were reported for several meta-GGA functionals, this behavior could not be reproduced from the reconstructed data in the present analysis.

Elayan and co-workers^56^ investigated 18 DIPY chromophores, assessing CAM-B3LYP, ωB97X,^85^ M06-2X,^78^ M11,^86^ and MN15 ^79^ against CC2 for excitation energies, dipole moments, and 2PA cross sections. The comparison relied primarily on MAEs, and the authors found that the TDDFT errors depended strongly on the chemical regime: the cross sections of the parent and halogen-substituted compounds were generally underestimated relative to CC2, whereas several derivatives were substantially overestimated.

Naim and co-workers^28^ constructed the most extensive small-molecule benchmark for 2PA strengths to date. The set comprises 25 molecules containing one to six non-hydrogen atoms and includes 82 transitions of valence or Rydberg character, together with several states having significant double-excitation contributions. CC3 was used as the reference, and five TDDFT approaches—B3LYP, BH&HLYP,^69,70^ CAM-B3LYP, and two LC-BLYP variants^74^—were assessed. The reported statistics included MAPE, MAE, and raw-scale *r* ^2^ values.

It was subsequently found that the CC3 reference values reported in the length gauge had been compared with TDDFT results obtained in the velocity gauge.^87^ To ensure a consistent comparison, we recalculated the TDDFT results in the length gauge using the Dalton program.^88^ The recalculations retained the molecular geometries, exchange–correlation functionals, basis sets, response formulation, and numerical settings of the original TDDFT protocol; only the gauge used to evaluate the 2PA strengths was changed. The revised benchmark data are provided both in the Supporting Information and in the associated Zenodo repository, and the complete Dalton output files are archived in the Zenodo repository.^68^ The discussion in the present work is based exclusively on these gauge-consistent results. A detailed investigation of the origin and magnitude of the discrepancies between the length- and velocity-gauge data is beyond the scope of the present study and is left to the original authors.

In an earlier study, Beerepoot and co-workers^51^ considered six four-coordinate organoboron chelates. PBE, BLYP, B3LYP, PBE0, LC-BLYP, and CAM-B3LYP were assessed against CC2, and the comparison relied mainly on direct ratios. This set provides a compact and chemically well-defined example of the distinction between quantitative agreement and reliable trend reproduction.

## 4 Discussion

### 4.1 Dipolar Push–Pull Chromophores

Among the benchmark sets considered here, the dipolar push–pull chromophore set of ref 40 is particularly well suited for comparing statistical descriptions. It contains 48 chemically related systems, spans several orders of magnitude in reference 2PA strength, and has been examined with a broad range of density functionals in subsequent studies.^41,57,58^ This combination of chemical coherence, wide dynamic range, and extensive functional coverage provides a strong basis for contrasting raw-scale and scale-invariant metrics. The results are summarized in Table 2.

**Table 2:** Statistical Results for the Dipolar Push–Pull Chromophore Benchmark (48 Transitions from 48 Molecules)^a^

functional	*B*	*F*	*F*_0.9_	ρ_rank_	ρ_*c*_	*b*_log_	MAE	*r* ^2^
BLYP	1.653	2.240	4.729	0.770	0.678	0.777	55301	0.850
B3LYP	1.127	1.628	3.144	0.864	0.839	0.872	31120	0.899
CAM-B3LYP	0.442	2.263	3.354	0.928	0.740	0.925	50112	0.993
LC-BLYP	0.276	3.627	5.117	0.956	0.552	0.881	68038	0.948
PBE	1.634	2.208	4.385	0.776	0.689	0.784	54600	0.852
PBE0	0.981	1.486	2.560	0.888	0.875	0.908	25824	0.906
OT-LC-BLYP	0.361	2.773	3.833	0.959	0.668	0.911	60415	0.964
cTPSSh	1.348	1.861	3.963	0.846	0.783	0.835	39893	0.889
cM06-2X	0.495	2.019	2.780	0.953	0.806	0.984	41236	0.984
cSCAN0	0.816	1.541	2.640	0.867	0.850	0.925	25263	0.905
cMN15	0.619	1.667	2.616	0.923	0.856	0.958	28085	0.949
cM06	0.844	1.552	2.678	0.865	0.851	0.913	25754	0.903
cBMK	0.657	1.579	2.474	0.930	0.875	0.968	24552	0.944
cTPSS	1.590	2.159	4.197	0.786	0.701	0.789	51756	0.857
cLC-TMLYP	0.285	3.507	5.226	0.937	0.555	0.863	67258	0.942

a All metrics are dimensionless except MAE, which is reported in a.u.

PBE0 provides the most favorable overall multiplicative accuracy, with cBMK, cSCAN0, and cM06 forming a similarly competitive group. These methods also rank among the best performers according to the MAEs, indicating that, in this case, the conventional raw-scale analysis does not produce a qualitatively misleading ordering. Nevertheless, the scale dependence of the raw error becomes particularly apparent when the total absolute error is decomposed by reference intensity. Across the 15 methods considered in this benchmark, the five strongest transitions constitute only 10.4% of the data set yet contribute 42.5–65.5% of the total raw absolute error. By contrast, they account for only 6.5–13.1% of the total absolute log-fold error. Thus, although the raw MAE yields a broadly reasonable ranking of the leading methods, it implicitly assigns disproportionate weight to the strongest absorbers. For this benchmark, the logarithmic transformation instead substantially reduces the concentration of the total error in the highest-intensity transitions. In line with these findings, PBE0, cBMK, cMN15, cSCAN0, and cM06 also exhibit the strongest logarithmic concordance, further confirming their generally good agreement with the reference data.

The comparison between PBE0 and cBMK nevertheless reveals that this similar overall performance arises from different error structures. PBE0 is nearly unbiased on the geometric-mean scale and gives the smallest typical fold error, but its *b*_log_ < 1 indicates a moderate compression of the dynamic range. cBMK shows a larger systematic underestimation, yet it combines low fold errors and favorable upper-tail behavior with better rank preservation and a *b*_log_ value closer to unity. Thus, PBE0 reproduces the absolute scale more accurately, whereas cBMK follows the relative spread and ordering of the reference strengths more faithfully. Overall, these results support the commonly stated conclusion that global hybrid functionals provide comparatively accurate absolute 2PA strengths while also showing that similar MAE or concordance values can arise from substantially different combinations of bias, tail behavior, dynamic-range reproduction, and rank preservation.

The bias dominance index provides an additional distinction between these error structures. To illustrate qualitatively different regimes without repeating the full 15-functional comparison, we selected five representative methods: PBE0 as a nearly unbiased case; cBMK and cMN15 as strongly bias-dominated but not fully one-sided cases; and CAM-B3LYP and cM06-2X as nearly one-sided underestimating cases. Because *B* and *F* are already reported in Table 2, only the additional diagnostics are listed in Table 3.

**Table 3:** Bias-Dominance Analysis for Five Functionals Selected to Represent Nearly Unbiased, Strongly Bias-Dominated but Not Fully One-Sided, and Nearly One-Sided Error Structures^a^

functional	*S*	fraction underestimated
PBE0	0.049	0.396
cBMK	0.920	0.917
cMN15	0.939	0.938
CAM-B3LYP	1.000	0.979
cM06-2X	1.000	1.000

a All values are based on 48 transitions from 48 molecules.

For CAM-B3LYP and cM06-2X, *S* is essentially unity, consistent with the observation that nearly all individual predictions underestimate the reference values. Their comparatively large fold errors therefore arise mainly from a coherent downward shift rather than from a broad two-sided error distribution. cBMK and cMN15 show a similar, although slightly less extreme, bias-dominated behavior. PBE0 represents the opposite limit: its *B* value is close to unity, whereas *F* = 1.486, resulting in *S* = 0.049. Thus, its small net bias arises from substantial cancellation between positive and negative logarithmic errors rather than from uniformly accurate predictions.

The LOO calibration was used to quantify how much of the systematic multiplicative shift can be removed without using the held-out transition. Table 4 reports results for five representative methods for which calibration produces a pronounced reduction in *F*, together with PBE0 as a nearly unbiased control. Because the uncalibrated factors are already given in Table 2, only *F*_LOO_ and its ratio to the original *F* are listed.

**Table 4:** LOO-Calibrated Fold-Error Factors for Five Representative Functionals Showing Pronounced Calibration Benefits, with PBE0 Included as a Nearly Unbiased Control^a^

functional	*F*_LOO_	FLOO/F
OT-LC-BLYP	1.221	0.440
LC-BLYP	1.228	0.339
cM06-2X	1.262	0.625
cLC-TMLYP	1.301	0.371
CAM-B3LYP	1.330	0.588
PBE0	1.505	1.013

a All values are based on 48 transitions from 48 molecules.

The improvement is particularly pronounced for the bias-dominated functionals including several range-separated approaches. The fold error factor decreases from 3.627 to 1.228 for LC-BLYP and from 2.263 to 1.330 for CAM-B3LYP. A similarly large improvement is obtained for cM06-2X, whose *F* value decreases from 2.019 to 1.262. In contrast, the nearly unbiased PBE0 results do not benefit from the correction, and its cross-validated fold error increases slightly from 1.486 to 1.505.

The results also reveal a systematic bias–ranking trade-off within the BLYP-based sequence (see Table 2). From BLYP through B3LYP and CAM-B3LYP to LC-BLYP, the rank correlation increases monotonically from 0.770 to 0.956, while the geometric mean bias changes from substantial overestimation, *B* = 1.653, to severe underestimation, *B* = 0.276. The functionals therefore move along a bias–ranking trade-off rather than following a single overall accuracy hierarchy.

The range-separated functionals display a markedly different error pattern. They give high rank correlations and generally reproduce relative trends well but systematically underestimate the absolute transition strengths. Their performance is therefore more favorable for ordering molecules than for uncalibrated quantitative prediction. CAM-B3LYP illustrates this behavior: its high ρ_rank_ and comparatively favorable *b*_log_ indicate good reproduction of the ordering and relative spread, while its low *B* and elevated *F* and *F*_0.9_ reflect a pronounced systematic underestimation of the absolute magnitude. This assessment is fully consistent with the raw-scale evaluation and does not lead to a different qualitative conclusion.

An interesting discrepancy emerges when the relationship between the raw-scale *r* ^2^ and the log–log slope *b*_log_ is examined in more detail. First, we were unable to reproduce the *r* ^2^ values reported in ref 57. Reconstructed data are available in ref 58 for the conventional, gauge-dependent M06-2X and MN15 calculations. Using the raw-scale *r* ^2^ definition given above, we obtain *r* ^2^ = 0.981 and 0.947 for M06-2X and MN15, respectively, whereas the original study reported *r* ^2^ = 0.995 for both functionals. For M06-2X, neither the alternative aug-cc-pVDZ data (*r* ^2^ = 0.986) nor standard alternative *R* ^2^ definitions reproduce the reported value. Consequently, conclusions regarding the superiority of the examined meta-GGA functionals that rely strongly on these correlations become uncertain. Based on our results, CAM-B3LYP and OT-LC-BLYP are among the highest-ranked methods according to *r* ^2^, together with cM06-2X, whereas several other meta-GGA functionals occupy only intermediate positions.

The ordering obtained from *b*_log_ is substantially different. cM06-2X and cBMK are among the best methods for reproducing the dynamic range, followed by cMN15, whereas the *r* ^2^ ranking gives much greater prominence to CAM-B3LYP and OT-LC-BLYP. This discrepancy arises because *r* ^2^ measures the tightness of a linear relationship on the raw scale, while *b*_log_ measures whether the relative separation between weak and strong transitions is reproduced correctly. cM06-2X illustrates this distinction particularly clearly: its high ρ_rank_ and near-ideal *b*_log_ indicate good reproduction of the ordering and dynamic range, while its low *B* reveals a largely systematic downward shift. This structured error pattern, which may permit an effective multiplicative calibration within the present benchmark set, cannot be identified from MAE or *r* ^2^ alone.

### 4.2 Dipyrromethene Chromophores

The statistical results for the DIPY benchmark^56^ are collected in Table 5.

**Table 5:** Statistical Results for the DIPY Benchmark (18 Transitions from 18 Molecules)^a^

functional	*n*	*B*	*F*	*F*_0.8_	ρ_rank_	ρ_*c*_	*b*_log_	MAE	*r* ^2^
CAM-B3LYP	18	0.682	1.745	1.946	0.987	0.902	1.286	454	0.931
ωB97X	18	0.835	1.578	1.703	0.990	0.923	1.205	471	0.911
M06-2X	18	0.555	2.168	2.343	0.838	0.776	1.308	508	0.925
M11	18	0.828	1.604	1.706	0.990	0.920	1.219	505	0.907
MN15	18	0.548	2.256	2.445	0.831	0.789	1.379	545	0.926

a All metrics are dimensionless except MAE, which is reported in a.u.

The raw MAE slightly favors CAM-B3LYP, whereas the scale-invariant metrics identify ωB97X and M11 as the most balanced methods. The latter two show nearly identical systematic bias, fold errors, logarithmic concordance, and rank preservation, indicating very similar overall error structures. Their favorable *B*, *F*, and *F*_0.8_ values are notable for range-separated functionals. An MAE-based ranking would nevertheless place CAM-B3LYP first and would make the difference between ωB97X and M11 appear larger than suggested by the scale-invariant metrics while also overlooking the stronger systematic underestimation of CAM-B3LYP.

M06-2X and MN15 exhibit larger multiplicative errors and weaker rank preservation than ωB97X and M11 despite retaining relatively high raw-scale *r* ^2^ values. This result is particularly notable because global hybrid functionals have generally been considered comparatively reliable for reproducing the absolute magnitude of 2PA strengths. At the same time, it illustrates why a high correlation on the original scale is not equivalent to good quantitative agreement: M11 has the lowest *r* ^2^ among the five functionals while sharing the highest rank correlation with ωB97X and giving very similar multiplicative accuracy.

All investigated functionals give *b*_log_ > 1, indicating an overestimation of the dynamic range. The interpretation of the global slope is, however, complicated by the presence of two chemically distinct regimes. As shown in Table 6, the geometric mean bias is systematically larger for the phenyl-substituted chromophores than for the parent and halogen-substituted compounds.

**Table 6:** Chemical-Regime Dependence of the Multiplicative Bias and log–log Calibration Slope in the DIPY Benchmark^a^

	parent/halogen subs			phenyl subs		
Functional	*n*	*B*	*b*_log_	*n*	*B*	*b*_log_
CAM-B3LYP	10	0.547	0.814	8	0.898	1.487
ωB97X	10	0.694	1.059	8	1.052	1.214
M06-2X	10	0.490	0.752	8	0.647	2.041
M11	10	0.682	0.971	8	1.056	1.251
MN15	10	0.434	0.685	8	0.735	1.908

a The parent/halogen and phenyl subsets contain 10 transitions from 10 molecules and 8 transitions from 8 molecules, respectively.

The subgroup-specific log–log slopes also differ substantially and, for several functionals, lie on opposite sides of unity. The global *b*_log_ > 1 therefore does not reflect only a uniform exaggeration of the dynamic range. This observation further illustrates the strong system dependence of TDDFT performance and suggests that robust, transferable conclusions across chemically distinct classes may be difficult to obtain.

### 4.3 Small Molecules

The small-molecule benchmark of ref 28 provides an opportunity to examine functional performance across chemically and electronically distinct classes of transitions using CC3 reference values. The data were therefore divided into physically motivated subsets according to the excitation character and magnitude of the 2PA strength. We retain the same partitioning to facilitate a direct comparison between the conventional raw-scale indicators and the proposed scale-invariant metrics. States with substantial double-excitation character, ambiguous assignments, or resonance-related cases were likewise excluded. For pyrrole, the closely spaced 1*B*_1_ and 2*B*_1_ transitions are strongly mixed. Following the original benchmark, their 2PA strengths were therefore summed, and the resulting value was treated as a single data point for the statistical analysis. The analysis further included only transitions for which both the reference and the corresponding TDDFT 2PA strengths exceeded 3.0 au, extending the reference-based threshold used previously. As a sensitivity check, we also repeated the analysis on the common intersection of 56 transitions retained for all five functionals after applying the same exclusion criteria, including the lower strength cutoff and the removal of states affected by problematic assignments or resonance-related complications. The corresponding results, including the same intensity and excitation-character subsets, are reported in Table S1 of the Supporting Information. The principal overall multiplicative conclusion is unchanged, with CAM-B3LYP remaining the most favorable method for the full benchmark, although some raw-scale and subgroup rankings depend on the precise composition of the set. It is also worth noting that the MAPE was already employed in the original study, thereby emphasizing the importance of a scale-independent assessment.

The statistical results are presented in Table 7.

**Table 7:** Statistical Results for the Small-Molecule Benchmark^a^

functional	*n*	*m*	*B*	*F*	*F*_0.9_	ρ_rank_	ρ_*c*_	*b*_log_	MAE	*r* ^2^
	all types of excitations									
B3LYP	69	23	1.161	1.368	1.998	0.948	0.935	0.948	19.4	0.971
BH&HLYP	68	23	0.921	1.294	1.705	0.947	0.948	1.028	13.7	0.915
CAM-B3LYP	66	23	1.002	1.189	1.365	0.968	0.975	0.957	13.9	0.882
LC-BLYP47	67	23	0.898	1.371	1.897	0.946	0.942	0.902	21.2	0.845
LC-BLYP33	64	23	0.963	1.266	1.688	0.955	0.949	0.966	16.1	0.875
	3 < δ^2PA^ < 20									
B3LYP	22	16	1.262	1.542	2.930	0.556	0.475	0.750	5.4	0.247
BH&HLYP	22	15	0.834	1.463	1.864	0.717	0.644	0.986	4.0	0.388
CAM-B3LYP	20	14	1.049	1.169	1.345	0.921	0.888	1.026	2.1	0.608
LC-BLYP47	21	13	1.004	1.549	2.114	0.534	0.574	0.716	4.7	0.295
LC-BLYP33	19	13	0.985	1.341	1.953	0.684	0.599	0.873	3.4	0.295
	20 ≤ δ^2PA^ < 80									
B3LYP	25	15	1.100	1.341	1.795	0.700	0.591	0.759	11.7	0.536
BH&HLYP	25	15	1.004	1.220	1.461	0.794	0.760	0.903	8.5	0.718
CAM-B3LYP	25	15	1.013	1.204	1.369	0.776	0.842	1.001	7.8	0.780
LC-BLYP47	24	15	0.917	1.298	1.612	0.803	0.789	1.145	11.3	0.660
LC-BLYP33	23	14	1.002	1.281	1.625	0.821	0.775	1.309	10.0	0.740
	80 ≤ δ^2PA^									
B3LYP	22	14	1.137	1.241	1.676	0.754	0.876	1.043	42.1	0.965
BH&HLYP	21	13	0.921	1.220	1.417	0.847	0.742	0.924	30.1	0.878
CAM-B3LYP	21	14	0.945	1.189	1.348	0.905	0.826	0.810	32.3	0.808
LC-BLYP47	22	14	0.789	1.295	1.720	0.861	0.715	0.725	47.7	0.801
LC-BLYP33	22	14	0.907	1.189	1.469	0.895	0.828	0.763	33.3	0.813
	valence excitations									
B3LYP	19	14	1.042	1.435	2.940	0.867	0.856	0.783	9.8	0.754
BH&HLYP	19	13	0.988	1.405	1.861	0.840	0.886	0.934	12.6	0.530
CAM-B3LYP	19	14	0.977	1.318	1.895	0.888	0.915	0.849	10.2	0.637
LC-BLYP47	21	14	1.005	1.495	2.370	0.845	0.870	0.820	11.0	0.617
LC-BLYP33	19	14	0.965	1.332	2.037	0.861	0.903	0.863	9.6	0.687
	Rydberg excitations									
B3LYP	49	21	1.210	1.346	1.928	0.962	0.940	0.959	21.5	0.970
BH&HLYP	49	21	0.896	1.253	1.704	0.974	0.960	1.072	14.1	0.928
CAM-B3LYP	46	21	1.032	1.121	1.244	0.991	0.991	1.007	9.4	0.980
LC-BLYP47	45	21	0.870	1.295	1.713	0.964	0.960	0.973	19.3	0.918
LC-BLYP33	44	20	0.983	1.219	1.513	0.974	0.958	1.027	12.4	0.971

a All metrics are dimensionless except MAE, which is reported in a.u. Here, *n* and *m* denote the numbers of retained transitions and represented molecules, respectively.

The comparison between the conventional raw-scale indicators and the scale-invariant metrics leads to markedly different impressions of functional performance. For the complete data set, the raw MAE identifies BH&HLYP as the best method, whereas the raw-scale *r* ^2^ strongly favors B3LYP. Neither conclusion is supported by the multiplicative analysis, for which CAM-B3LYP provides the most favorable overall multiplicative accuracy. CAM-B3LYP has an essentially negligible geometric-mean bias (*B* = 1.002), the smallest typical fold error (*F* = 1.189), the smallest 90th-percentile fold error (*F*_0.9_ = 1.365), and the highest concordance and rank correlation. B3LYP, although characterized by an excellent raw-scale correlation (*r* ^2^ = 0.971), systematically overestimates the reference strengths (*B* = 1.161) and exhibits substantially broader typical and upper-tail errors. Despite its high raw-scale correlation, B3LYP does not yield a comparably favorable log–log slope (*b*_log_ = 0.948), as might be expected if the dynamic range were reproduced equally well. Its high *r* ^2^ therefore mainly reflects the overall large-scale variation in the data rather than superior reproduction of the dynamic range or more accurate predictions of individual 2PA strengths.

The origin of this discrepancy becomes clear when the benchmark is divided by intensity. For CAM-B3LYP, the raw MAE rises from 2.1 a.u. for the weakest transitions to 32.3 a.u. for the strongest ones, whereas the corresponding fold errors remain almost unchanged, with *F* = 1.169 and 1.189, respectively. Thus, the approximately 15-fold increase in MAE primarily reflects the change in the absolute scale of the reference values rather than a deterioration of the relative predictive accuracy. The same issue affects the raw-scale *r* ^2^. B3LYP yields *r* ^2^ = 0.971 for the full benchmark but only 0.247 for the weakest and 0.536 for the intermediate-intensity transitions. Its apparently excellent overall correlation is consequently generated to a large extent by combining subsets with widely separated magnitudes and by the high leverage of the largest responses. In contrast, the log–log slope *b*_log_ remains considerably more balanced across the subsets, because it characterizes the relative expansion or compression of the predicted dynamic range rather than the raw-scale separation of the data.

For the strongest transitions, the raw and scale-invariant rankings partly converge but still describe different properties. B3LYP reproduces the overall raw-scale variation well but retains a positive multiplicative bias and a weaker rank correlation than CAM-B3LYP. Conversely, the range-separated functionals show *b*_log_ < 1, indicating compression of the relative dynamic range even when their rank correlations remain favorable. Hence, a high *r* ^2^ does not ensure that the relative spacing of the 2PA strengths is reproduced, whereas a small MAE does not distinguish whether an error is large or small relative to the transition intensity.

The excitation-type subsets reinforce the same conclusion. For Rydberg states, CAM-B3LYP gives the most favorable performance according to both the raw MAE and the scale-invariant analysis. Its geometric-mean bias is close to unity, and it exhibits the smallest typical and 90th-percentile fold errors, together with the highest concordance and rank correlation. Although B3LYP also yields a high raw-scale *r* ^2^, its larger positive bias and broader fold-error distribution show that this favorable correlation does not translate into equally accurate predictions of individual 2PA strengths. For the valence subset, the raw indicators are more ambiguous: LC-BLYP33 has the smallest MAE, whereas B3LYP has the largest *r* ^2^. The scale-invariant metrics instead show that CAM-B3LYP has the smallest typical fold error and the best rank and concordance coefficients, while BH&HLYP has the most favorable log–log slope and upper-tail factor.

### 4.4 Organoboron Chelates

For the organoboron benchmark of ref 51, the transitions were matched using the reported state assignments rather than their energetic ordering alone. Consistent with the original benchmark, we also omitted molecule 5 because of its ambiguous state assignment. Although the benchmark comprises only ten transitions for five molecular systems, it nevertheless reveals informative differences among the statistical measures and functional classes. The statistical results are collected in Table 8.

**Table 8:** Statistical Results for the Organoboron Benchmark (10 Transitions from 5 Molecules)^a^

functional	*n*	*B*	*F*	*F*_0.8_	ρ_rank_	ρ_*c*_	*b*_log_	MAE	*r* ^2^
CAM-B3LYP	10	0.486	2.059	2.630	0.806	0.368	0.439	2653	0.935
LC-BLYP	10	0.322	3.105	3.830	0.745	0.285	0.620	3149	0.919
B3LYP	10	0.820	1.373	1.502	0.782	0.671	0.479	1743	0.592
BLYP	10	0.824	1.702	2.332	0.782	0.620	0.744	1996	0.440
PBE	10	0.756	1.746	2.488	0.745	0.600	0.828	1947	0.500
PBE0	10	0.736	1.427	1.604	0.794	0.653	0.518	1830	0.654

a All metrics are dimensionless except MAE, which is reported in a.u.

The raw-scale analysis provides only a partial description of the error structure for this benchmark. The MAEs favor the global hybrid functionals, yielding values between approximately 1750 and 1850 au, whereas CAM-B3LYP and LC-BLYP give larger deviations of 2653 and 3149 au, respectively. The raw-scale *r* ^2^ values lead to a markedly different impression. CAM-B3LYP and LC-BLYP exhibit the strongest linear correlations, with *r* ^2^ = 0.935 and 0.919, while the corresponding values for the global hybrid functionals range from 0.592 to 0.654. Thus, the conventional metrics favor different functional classes depending on whether absolute deviations or raw-scale linear association are considered.

The scale-invariant metrics reveal that these apparently conflicting rankings originate from different error structures. B3LYP and PBE0 provide the most favorable overall multiplicative accuracy. B3LYP has the smallest typical and upper-tail fold errors, *F* = 1.373 and *F*_0.8_ = 1.502, together with the highest logarithmic concordance, ρ_c_ = 0.671. PBE0 gives closely comparable values. Their geometric mean biases, *B* = 0.820 and 0.736, respectively, indicate moderate systematic underestimation, but their individual predictions remain substantially closer to the reference values than suggested by their only moderate raw-scale *r* ^2^ values. Their rank preservation is also competitive with that of the range-separated hybrids: B3LYP and PBE0 yield ρ_rank_ = 0.782 and 0.794, respectively, compared with 0.806 for CAM-B3LYP and 0.745 for LC-BLYP. Thus, the superior multiplicative accuracy of the global hybrids is not obtained at the expense of substantially poorer ordering of the transitions.

A further distinction is obtained from the log–log slopes. All functionals yield *b*_log_ < 1, indicating compression of the dynamic range, but the effect is strongest for CAM-B3LYP, with *b*_log_ = 0.439. This is particularly notable because CAM-B3LYP simultaneously gives the largest raw-scale correlation, *r* ^2^ = 0.935. The combination shows that a very tight linear relationship on the original scale can coexist with a strongly compressed relative separation between weak and strong transitions. By contrast, BLYP and particularly PBE give slopes closer to unity, *b*_log_ = 0.744 and 0.828. Thus, the local functionals reproduce the relative dynamic range more faithfully, even though their fold errors and logarithmic concordance are less favorable than those of the best global hybrids and their raw-scale correlations are much weaker than those of the range-separated hybrids.

## 5 Conclusions

In this study, a scale-invariant framework has been introduced for the statistical assessment of TDDFT 2PA strengths. To this end, the conventional raw-scale analysis was complemented by metrics derived from logarithmic fold errors. The geometric mean bias and the typical and high-percentile fold-error factors quantify the systematic and unsigned multiplicative deviations, respectively, while the logarithmic concordance coefficient, the log–log calibration slope, and the rank correlation characterize overall agreement, dynamic-range reproduction, and ordering. In addition, the bias-dominance index and the LOO-calibrated fold-error factor were employed to determine whether the errors primarily originate from a coherent multiplicative shift and whether such a shift can be removed without using the observation being predicted.

The consequences of the statistical description were first examined in detail for the benchmark comprising 48 dipolar push–pull chromophores.^40,41,57,58^ The five strongest transitions represent only 10.4% of the data set but account for 42.5–65.5% of the total raw absolute error for the functionals considered. By contrast, their contribution to the total absolute log-fold error is only 6.5–13.1%. Thus, even when the raw MAE produces a broadly reasonable ranking of the leading methods, it assigns disproportionate weight to a small number of intense transitions. The scale-invariant analysis also revealed that similar overall errors may arise from fundamentally different error structures. PBE0 is nearly unbiased and provides the smallest typical fold error, whereas cBMK combines a coherent underestimation with better rank preservation and dynamic-range reproduction. Several range-separated approaches exhibit even larger systematic underestimations, but their favorable rank correlations and the substantial improvements obtained after LOO calibration show that these errors contain a strong coherent multiplicative component.

The analyses of the remaining benchmark sets reinforce these conclusions. For the DIPY chromophores, the raw MAE slightly favors CAM-B3LYP, whereas the multiplicative statistics identify ωB97X and M11 as the most balanced approaches. The subgroup-specific results further demonstrate that a single global slope can mask markedly different error regimes for chemically distinct derivatives. For the small-molecule benchmark, CAM-B3LYP provides the most favorable overall multiplicative performance, despite the raw MAE and *r* ^2^ favoring BH&HLYP and B3LYP, respectively. In particular, the approximately 15-fold increase in the CAM-B3LYP MAE from the weakest to the strongest transitions is not accompanied by a deterioration of the fold error, confirming that the raw deviation mainly reflects the change in the numerical scale. The common-intersection sensitivity analysis preserves this overall CAM-B3LYP multiplicative ranking while showing that some raw-scale and subgroup rankings are more sensitive to the precise composition of the benchmark set. Finally, for the organoboron chelates, B3LYP and PBE0 deliver the best multiplicative accuracy, while the high raw-scale correlations of CAM-B3LYP and LC-BLYP coexist with a pronounced compression of the dynamic range. Because the underlying CC references are not of uniform accuracy, differences between benchmark sets and small numerical differences between functionals should not be overinterpreted.

Overall, the scale-invariant analysis largely supports the principal qualitative observations reported in the original benchmark studies but places them on a more transparent and quantitatively comparable basis. At the same time, it enables conclusions that cannot be obtained from MAEs and raw-scale linear regression statistics alone. In particular, neither an individual raw-scale metric nor a combination of such metrics is sufficient to identify a reliable or generally recommended functional. We therefore recommend that future benchmark studies report multiplicative bias, typical and upper tail fold errors, logarithmic concordance, dynamic range reproduction, and rank preservation as complementary quantities. Although demonstrated here for 2PA strengths, the proposed framework is directly applicable to other properties for which the errors are predominantly multiplicative and the reference values cover a broad dynamic range.

## Supplementary Material





## Data Availability

The benchmark data underlying this study, including the revised length-gauge TDDFT results, are provided in XLSX format in the Supporting Information and are also archived on Zenodo. The Python script used to calculate the statistical metrics and the complete Dalton output files are available in the same Zenodo repository.^68^

## Notes

Signed log-residual diagnostic for the dipolar push–pull chromophore benchmark and common-intersection sensitivity analysis for the small-molecule benchmark (PDF)
Compiled literature benchmark data and revised length-gauge TDDFT results (XLSX)
