# Supplementary material for: Multiplicative Error Analysis: A Scale-Invariant View of TDDFT Two-Photon Absorption Benchmarks
Source: J Chem Theory Comput. 2026 Oct 18;22(21):11378–92. doi: 10.1021/acs.jctc.6c01476 (PMC13757703; doi:10.1021/acs.jctc.6c01476)
Supplement: Supplementary file 1 [file ct6c01476_si_001.pdf]

Supporting Information: Multiplicative Error Analysis: A Scale-Invariant View of TDDFT Two-Photon Absorption Benchmarks

Dávid Mester[*,†,‡,¶] and Mihály Kállay[†,‡,¶]

†*Department of Physical Chemistry and Materials Science, Faculty of Chemical Technology and Biotechnology, Budapest University of Technology and Economics, Műegyetem rkp. 3., H-1111 Budapest, Hungary*

‡*HUN-REN–BME Quantum Chemistry Research Group, Műegyetem rkp. 3., H-1111 Budapest, Hungary*

¶*MTA–BME Lendület Quantum Chemistry Research Group, Műegyetem rkp. 3., H-1111 Budapest, Hungary*

E-mail: mester.david@vbk.bme.hu

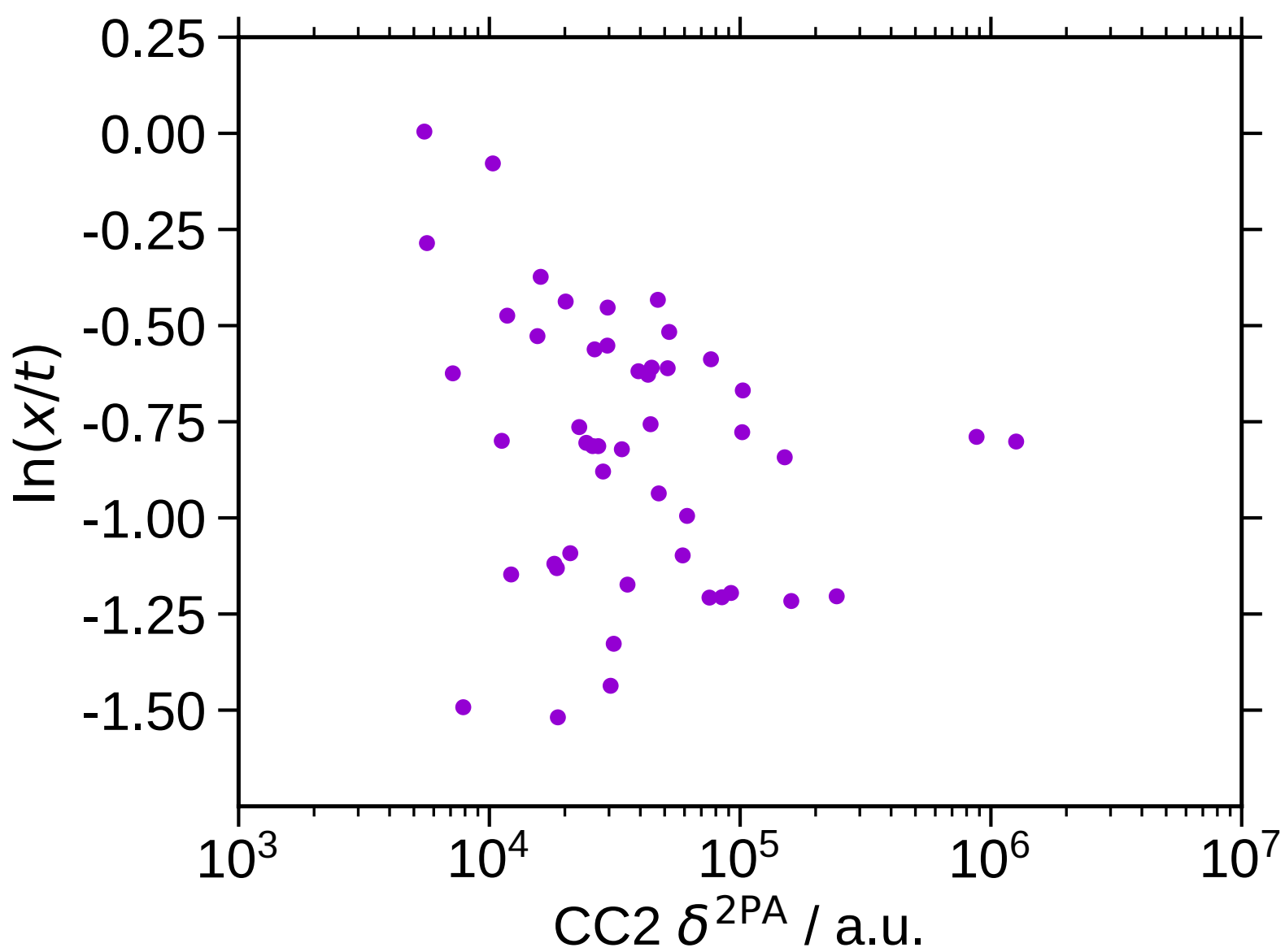

Figure S1: Signed logarithmic residuals, $\ln(x_i/t_i)$, for CAM-B3LYP on the 48-molecule dipolar push–pull chromophore benchmark, plotted against the CC2 reference 2PA strengths.

Table S1: Statistical results for the common-intersection sensitivity analysis of the small-molecule benchmark. All metrics are dimensionless except MAE, which is reported in a.u. Here n and m denote the numbers of retained transitions and represented molecules, respectively.

Functional	n	m	B	F	$F_{0.9}$	ρ_{rank}	ρ_c	$b_{\log}$	MAE	r^2
					All excitations					
B3LYP	56	23	1.105	1.326	1.835	0.948	0.943	0.977	18.939	0.967
BH&HLYP	56	23	0.949	1.253	1.529	0.946	0.956	0.999	15.618	0.913
CAM-B3LYP	56	23	1.004	1.175	1.365	0.967	0.978	0.987	10.627	0.969
LC-BLYP47	56	23	0.870	1.314	1.707	0.949	0.949	0.989	17.889	0.902
LC-BLYP33	56	23	0.991	1.210	1.508	0.960	0.969	0.984	12.220	0.963
					$3 < \delta^{2PA} < 20$					
B3LYP	14	12	1.133	1.437	2.158	0.609	0.579	0.812	4.450	0.445
BH&HLYP	14	12	0.885	1.274	1.614	0.843	0.819	1.029	2.293	0.699
CAM-B3LYP	14	12	1.003	1.132	1.315	0.899	0.928	0.904	1.229	0.892
LC-BLYP47	14	12	0.815	1.450	1.822	0.670	0.581	0.785	3.450	0.359
LC-BLYP33	14	12	1.024	1.153	1.243	0.942	0.931	1.008	1.650	0.819
					$20 \leq \delta^{2PA} < 80$					
B3LYP	22	14	1.081	1.352	1.747	0.682	0.602	0.726	11.182	0.597
BH&HLYP	22	14	1.020	1.263	1.503	0.756	0.746	0.883	9.818	0.705
CAM-B3LYP	22	14	1.020	1.230	1.446	0.775	0.831	0.974	8.523	0.768
LC-BLYP47	22	14	0.950	1.304	1.620	0.809	0.795	1.113	11.700	0.650
LC-BLYP33	22	14	1.013	1.308	1.636	0.825	0.766	1.283	10.759	0.734
					$80 \leq \delta^{2PA}$					
B3LYP	20	13	1.113	1.227	1.683	0.713	0.861	1.054	37.615	0.963
BH&HLYP	20	13	0.919	1.229	1.443	0.836	0.741	0.925	31.325	0.878
CAM-B3LYP	20	13	0.986	1.147	1.255	0.899	0.873	0.978	19.520	0.959
LC-BLYP47	20	13	0.827	1.238	1.579	0.872	0.770	0.852	34.805	0.911
LC-BLYP33	20	13	0.946	1.150	1.258	0.875	0.888	0.918	21.225	0.961
					Valence excitations					
B3LYP	16	12	0.913	1.335	1.693	0.897	0.904	0.909	9.688	0.758
BH&HLYP	16	12	0.910	1.368	1.596	0.832	0.879	0.986	12.925	0.519
CAM-B3LYP	16	12	0.910	1.298	1.575	0.876	0.913	0.856	10.619	0.631
LC-BLYP47	16	12	0.915	1.445	2.347	0.847	0.849	0.857	12.406	0.577
LC-BLYP33	16	12	0.915	1.253	1.688	0.891	0.930	0.863	9.606	0.693
					Rydberg excitations					
B3LYP	40	20	1.193	1.322	1.925	0.959	0.945	0.951	22.640	0.972
BH&HLYP	40	20	0.964	1.210	1.403	0.969	0.972	0.994	16.695	0.926
CAM-B3LYP	40	20	1.044	1.129	1.255	0.990	0.990	0.997	10.630	0.979
LC-BLYP47	40	20	0.853	1.265	1.650	0.966	0.968	1.031	20.083	0.917
LC-BLYP33	40	20	1.023	1.194	1.499	0.978	0.975	0.994	13.265	0.972
